# Assessing anatomical distribution of atopic dermatitis identifies a cluster of patients with late onset and low risk of asthma and allergy: An observational study

**DOI:** 10.1002/hsr2.1219

**Published:** 2023-05-04

**Authors:** Zarqa Ali, Kathryn Anderson, Anders D. Andersen, Priyanka Dahiya, John R. Zibert, Simon F. Thomsen

**Affiliations:** ^1^ Department of Dermato‐Venereology and Wound Healing Centre Copenhagen University Hospital Bispebjerg Copenhagen Denmark; ^2^ Studies&Me A/S Copenhagen Denmark; ^3^ Department of Biomedical Sciences University of Copenhagen Copenhagen Denmark

**Keywords:** allergy, asthma, aopic dermatitis, eczema, photographs

## Abstract

**Background and Aims:**

A better understanding of distinct subgroups in atopic dermatitis (AD) is warranted. The aim was to identify and determine characteristics of clusters based on anatomical location of AD.

**Methods:**

In this 8‐week, observational, decentralized study, patients with AD completed a baseline questionnaire about anatomical location and severity of AD, and a principal component analysis (PCA) was applied to identify clusters. The Patient‐Oriented Eczema Measure (POEM) was completed weekly and photographs of affected body areas were captured by the participants' own smartphones. From the weekly photographs, the AD severity was evaluated using the intensity part of the SCORing Atopic Dermatitis.

**Results:**

Fifty‐five participants were recruited, of which 53 completed the baseline questionnaire with a mean POEM of 14.5 (SD: 5.6). The PCA analysis revealed three clusters, with AD predominantly on the shins, knees, and genitals (Cluster 1), with involvement of the upper body (Cluster 2), and with AD on the hands and feet (Cluster 3). Cluster 1 had a lower mean POEM score (11.12, SD: 5.3) compared with Clusters 2 (12.64, SD: 4.5) and 3 (15.98, SD: 4.7), respectively (*p* = 0.007). Further, Cluster 1 had the highest age of AD onset (mean 9.5 vs. 2.5 and 4.7 years, *p* = 0.02) and the lowest proportion of asthma/allergy (47% vs. 82% and 90%, *p* = 0.01).

**Conclusion:**

Three clusters of patients with AD based on affected body areas were identified. The cluster with involvement of legs and genitals was characterized by the oldest age of AD onset and the lowest prevalence of asthma/allergy.

## 1 INTRODUCTION

Atopic dermatitis (AD) is a chronic, severely pruritic, skin disease that prominently reduces the quality of life of affected patients.^1^ AD is characterized by skin inflammation along with skin barrier dysfunction^2^ and affects different body areas depending on the age of the patient. The face and neck are commonly affected in infants and small children; flexural involvement is common in later childhood and adolescence, whereas in adults, the face, hands, and feet are more often affected.^3^ Common features seen in patients with AD are dry skin, early disease onset (<2 years), and a personal history and/or family history of atopic disease (e.g., AD, asthma, allergic rhinitis) or specific IgE reactivity.^4^ Early‐onset AD is the most common type; around 50% of all patients with AD develop symptoms within the first year of life and around 95% experience an onset below 5 years of age.^5^ Predictors of AD activity beyond childhood and into adulthood include concurrent asthma, allergic rhinitis, young age of onset, low socioeconomic status, and non‐White ethnicity.^6^, ^7^

AD is a heterogeneous disease and can be categorized in many phenotypes based on age of onset, disease severity and clinical or morphological features (eg. nummular eczema, atopic prurigo, lichen planus‐like, pityriasis alba).^8^ AD can also be categorized in extrinsic and intrinsic subtypes based on IgE levels and intrinsic AD is defined by female predominance and lack of association with respiratory atopy.^9^

To facilitate the development of successful prevention and treatment strategies, it is important to define the different phenotypes or subgroups of AD, and to evaluate the characteristics of these subgroups and associations with atopic diseases.

The aim of this study was to identify clusters of body areas affected by AD in adults, determine characteristics of these clusters based on age of onset and other atopic diseases, and to explore patterns of subjective and clinical changes, based on photographic assessments of AD lesions, in disease activity over time.

## 2 METHODS

The present study is a first‐time analysis of AD data from a previously published 8‐week, noninterventional, observational, fully remote decentralized feasibility study including patients with AD, designed to investigate whether a siteless trial could recruit nationwide, achieve high adherence, and prevent dropouts.^10^ Patients with AD were recruited online through advertisements on social media (recruitment data are published elsewhere^10^), and were included if they were 18 years or older, fulfilled the UK Diagnostic Criteria for Atopic Dermatitis,^11^ and had at least one visible AD lesion at the time of recruitment. The AD lesion was confirmed by board certified dermatologists on a photograph taken by the patient as the study was decentralized without visit to a clinical study site.

At baseline, the participants completed a questionnaire about body areas (scalp, face, neck, shoulders, arms, hands, chest, back, stomach, genitals, thighs, knees, shins, and feet) affected by AD and self‐reported AD severity at the affected body areas (none, mild‐to‐moderate, or severe). Throughout the study period, all study related tasks were performed from patients' own homes, as previously described.^10^ In brief, each week all participants received an email prompting them to capture a photograph of the affected skin and with a link to an online questionnaire (Google Forms). The Patient‐Oriented Eczema Measure (POEM),^12^ is a validated, reliable, and simple tool for measuring AD severity. The POEM scores can range from 0 to 28 and have shown longitudinal sensitivity to changes in activity of AD experienced by the patient.^12^ POEM items assess the frequency of itching, sleep disturbance, dryness, flaking, cracking, bleeding, and weeping/oozing because of AD during the past week.

POEM was completed weekly along with photographs of affected body areas captured by the participants' own smartphones. From POEM changes in dryness, flaking, cracking, bleeding, and weeping/oozing skin was extracted. SCORing Atopic Dermatitis (SCORAD) is a clinical tool to assess the severity of AD as an objective measure.^13^ From the photographs, the AD severity was evaluated using the intensity part of the SCORAD (iSCORAD). The iSCORAD is based on the ratings of the following six items: erythema, edema/papulation, excoriations, lichenification, oozing/crusts, and dryness, graded on a scale of 0–3.

## 3 ETHICS

Danish Regional Committee on Health Research Ethics was consulted before execution and the study did not require ethical approval (protocol number:16025688). The study was carried out according to Danish law.

### 3.1 Statistics

Baseline variables were reported as means and SDs for continuous variables and frequencies and percentages for categorical variables. A principal component analysis (PCA) was applied on the self‐reported body area locations and self‐reported severity given in the baseline questionnaire. Principal components were then clustered using the *k*‐means clustering in the statistical software R. The longitudinal POEM and iSCORAD data were analyzed for each of the three identified clusters and illustrated using box plots. Mean, SD, changes per observations, and slope of the longitudinal POEM and iSCORAD data was calculated.

The Kruskal–Wallis test by ranks and Pearson's *χ* ^2^ test along with Fisher's exact test was used to calculate two‐sided *p* values. A *p* < 0.05 was considered statistically significant.

All analyses were performed using the statistical software R.

## 4 RESULTS

**Figure 1:**
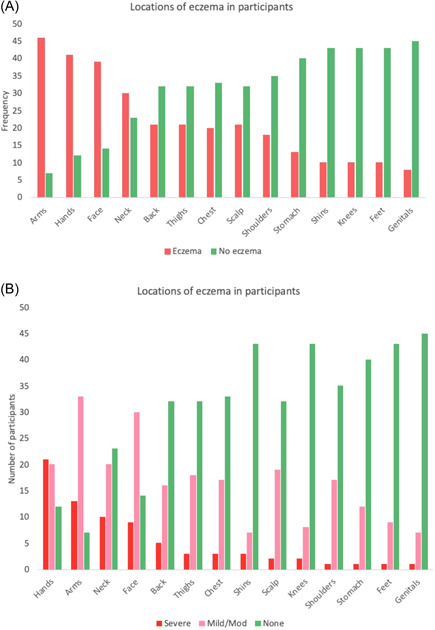
Self‐reported body areas with atopic dermatitis at baseline (A) and self‐reported severity in these areas (B).

Fifty‐five patients were recruited of which 53 completed the baseline questionnaire with a mean POEM of 14.5 (SD: 5.6) (range: 6–28). The body areas most often affected at baseline were the arms, hands, face, and neck, whereas the least often affected areas (reported as being without AD) were the stomach, genitals, knees, shins, and feet. The body areas most often reported as severe were the face, neck, arms and hands, whereas the body areas that were more commonly reported as mild‐to‐moderate were the scalp, face, neck, arms, hands, and thighs (Figure 1).

**Figure 2:**
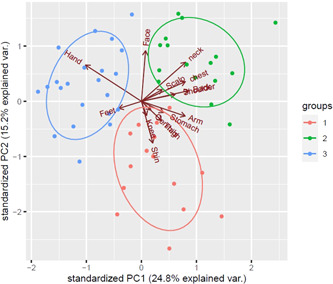
Principal component analysis on the body area scores.

The PCA analysis revealed three clusters corresponding to patients, respectively, as follows: AD predominantly on the shins, knees, and genitals (Cluster 1); on the upper body, especially the neck and face, shoulders, and arms (Cluster 2); and on the hands and feet (Cluster 3) (Figure 2).

**Figure 3:**
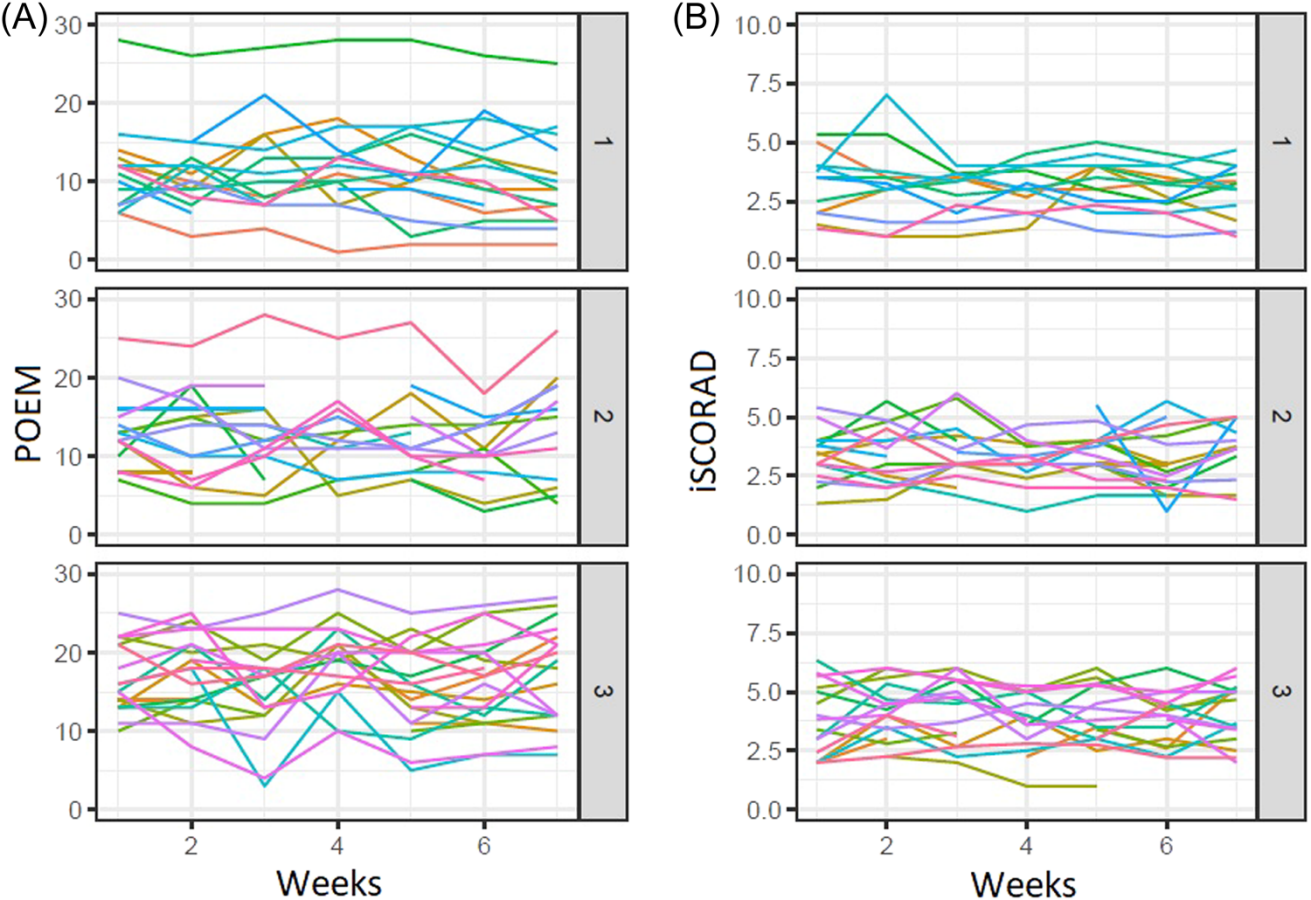
Eight‐week data for the Patient Oriented Eczema Measure (POEM) (A) and intensity part of the SCORing Atopic Dermatitis (iSCORAD) (B) data for the participants in the three clusters.

**Figure 4:**
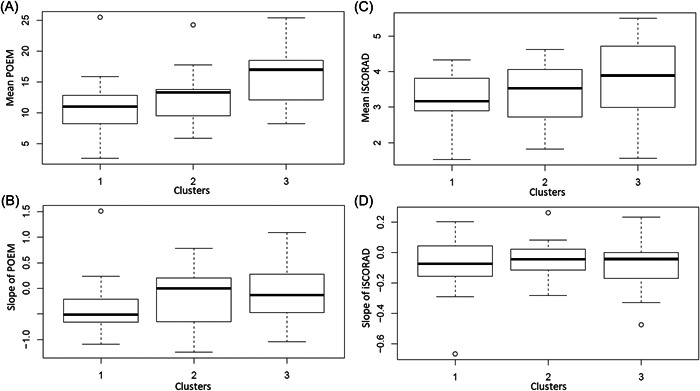
Mean data for the Patient Oriented Eczema Measure (POEM) (A) and slope of POEM (B) in all three clusters, and mean of intensity part of the SCORing Atopic Dermatitis (iSCORAD) (C) and slope of iSCORAD (D) in all three clusters.

Analysis of longitudinal POEM data throughout the 8‐week period (Figure 3A) showed that Cluster 1 had statistically significant (*p* = 0.007) lower mean POEM score (11.12, SD: 5.3) compared with Clusters 2 (12.64, SD: 4.5) and 3 (15.98, SD: 4.7), respectively (Figure 4A). Further, in Cluster 1 there appeared to be an improvement in AD severity overtime, as indicated by the negative slope of POEM (Figure 4B), with less fluctuation measured in changes per observation (Table 1). The reduction in POEM scores in Cluster 1 was driven by improvement of itch and sleep, which were the only elements with a decline in slope; however, these changes within Cluster 1 over time were not statistically significant and neither was the difference in slope between the clusters (Table 1). No clear change in self‐assessed AD activity in terms of changes in skin symptoms (as indicated with the slope estimates for bleeding, weeping/oozing, cracked, flaking, or dry skin) (Table 1) was observed. This was in line with clinical signs measured with iSCORAD. Analysis of longitudinal iSCORAD data throughout the 8‐week period (Figure 3B) showed that the mean iSCORAD was lowest in Cluster 1 (3.13, SD: 0.90) compared with Clusters 2 (3.34, SD: 0.90) and 3 (3.80, SD: 1.09); however, this was not statistically significant (Figure 4C). Further, no significant differences were seen in the change (slope) of iSCORAD (Figure 4D).

**Table 1:** Changes in AD during the 8‐week study period divided into the three clusters.

	Cluster 1	Cluster 2	Cluster 3	*p*
POEM				
Mean (SD)	11.12 (5.3)	12.64 (4.5)	15.98 (4.7)	0.007^§^
Slope	−0.32 (0.62)	−0.18 (0.60)	−0.07 (0.60)	0.3
Changes per observation	0.46 (0.16)	0.41 (0.21)	0.46 (0.13)	0.5
Itch skin				
Mean (SD)	2.62 (0.81)	2.76 (1.10)	3.08 (0.86)	0.3
Slope	−0.07 (0.13)	−0.04 (0.11)	−0.05 (0.16)	0.4
Changes per observation	0.31 (0.17)	0.37 (0.23)	0.30 (0.25)	0.5
Sleep disturbance				
Mean (SD)	0.79 (0.93)	0.98 (1.07)	0.95 (0.54)	0.3
Slope	−0.05 (0.11)	−0.05 (0.12)	0.00 (0.14)	0.3
Changes per observation	0.27 (0.21)	0.25 (0.22)	0.30 (0.22)	0.8
Bleeding skin				
Mean (SD)	0.83 (0.96)	1.02 (0.97)	1.35 (0.95)	0.2
Slope	−0.06 (0.15)	0.01 (0.15)	0.01 (0.11)	0.2
Changes per observation	0.19 (0.18)	0.26 (0.22)	0.38 (0.20)	0.03^§^
Weeping or oozing skin				
Mean (SD)	0.56 (0.99)	0.67 (0.91)	1.48 (1.04)	0.001^§^
Slope	0.00 (0.08)	0.01 (0.09)	0.01 (0.12)	0.5
Changes per observation	0.12 (0.18)	0.22 (0.25)	0.41 (0.21)	<0.001^§^
Cracked skin				
Mean (SD)	1.05 (1.21)	1.37 (1.04)	2.38 (1.18)	0.004^§^
Slope	−0.05 (0.18)	0.02 (0.18)	0.02 (0.18)	0.5
Changes per observation	0.23 (0.20)	0.28 (0.20)	0.26 (0.18)	0.8
Flaking skin				
Mean (SD)	2.04 (1.29)	2.65 (1.02)	3.02 (0.95)	0.05^§^
Slope	−0.06 (0.19)	−0.05 (0.19)	−0.03 (0.18)	0.6
Changes per observation	0.33 (0.23)	0.27 (0.18)	0.24 (0.19)	0.4
Dry skin				
Mean (SD)	3.22 (0.97)	3.19 (0.91)	3.72 (0.54)	0.06
Slope	−0.03 (0.19)	−0.08 (0.14)	−0.02 (0.09)	0.4
Changes per observation	0.22 (0.25)	0.22 (0.19)	0.11 (0.18)	0.1
iSCORAD				
Mean (SD)	3.13 (0.90)	3.34 (0.90)	3.80 (1.09)	0.2
Slope	−0.09 (0.21)	−0.04 (0.12)	−0.08 (0.17)	0.8
Changes per observation	0.47 (0.17)	0.44 (0.11)	0.51 (0.17)	0.2

*Note*: ^§^denotes statistically significant *p* values.

Abbreviations: AD, atopic dermatitis; iSCORAD, intensity part of the SCORing Atopic Dermatitis; POEM, Patient‐Oriented Eczema Measure.

Cluster 1 had a significant lower proportion of participants with early (<2 years) disease onset (20%) compared with Clusters 2 (65%) and 3 (57%) (*p* = 0.02). Further, the percentage of participants with other atopic diseases (asthma or allergy) was also lowest in Cluster 1 (47%) compared with Clusters 2 (82%) and 3 (90%) (*p* = 0.01). A tendency was observed toward lower use of moisturizers, systemic treatment, and phototherapy for AD at baseline in Cluster 1 (Table 2).

**Table 2:** Characteristics of patients in the three clusters.

	Cluster 1 *N* = 15	Cluster 2 *N* = 17	Cluster 3 *N* = 21	*p*
Sex, female/male, *n* (%)	13 (87)/2 (13)	14 (82)/3 (18)	21 (100)/0 (0)	0.1
Age, years, mead (SD)	27.5 (8.37)	29.7 (11.1)	28.1 (9.48)	0.9
Age of onset, years, mean (SD)	9.5 (12.8)	2.47 (3.10)	4.67 (6.72)	0.02^a^
Age of onset median (IQR)	5.0 (3.0‐9.0)	2.0 (0.0‐3.0)	2.0 (1.0‐5.0)	0.02^a^
Age of onset (<2 years), *n* (%)	3 (20)	11 (65)	12 (57)	0.6
Age of onset (<5 years), *n* (%)	11 (73)	15 (88)	17 (80)	
Asthma or allergy, *n* (%)	7 (47)	14 (82)	19 (90)	0.01^a^
Daily moisturizer^b^, *n* (%)	6 (40)	12 (71)	17 (81)	0.03^a^
Moisturizer at least every other day^b^, *n* (%)	12 (80)	17 (100)	20 (95)	0.09
Topical treatment^b^, *n* (%)	7 (47)	13 (76)	17 (80)	0.08
Systemic treatment^b^, *n* (%)	1 (7)	2 (12)	1 (5)	0.8
Phototherapy^b^, *n* (%)	0 (0)	3 (18)	2 (10)	0.8

Abbreviation: IQR, interquartile range.

^a^ Statistically significant.

^b^ At baseline.

## 5 DISCUSSION

In this decentralized study with remotely collected patient reported data, three clusters of patients with AD based on anatomical distribution of eczema were identified: the first cluster was characterized by AD on legs and genitals, the second was characterized by involvement of the upper body, whereas the third was described by AD on the hands and feet. The patients with involvement of legs and genitals experienced most subjective improvement based on POEM throughout the 8‐week study period. However, the improvement was not reflected in iSCORAD. Further, this group had the highest age of AD onset and the lowest prevalence of asthma and allergy.

The mean POEM and iSCORAD scores followed each other and were lowest for Cluster 1. The low subjective and clinically assessed severity of AD in patients of Cluster 1 may explain the low usage of daily moisturizers and systemic treatment in this group. Further, a decline in POEM was observed in Cluster 1 patients but this was not reflected in objectively assessed iSCORAD data. This could indicate that the subjectively felt changes (e.g., itch) in disease activity precede the objective skin changes. The changes in iSCORAD might have been visible if the study duration was longer.

Moreover, Cluster 1 had the lowest proportion of patients with onset of AD before the age 2 years and at the same time they had the lowest prevalence of asthma and allergy. This is in line with a study by Wan et al.^14^ showing that early‐onset AD is associated with a greater risk of seasonal allergy and asthma than later onset AD. The risk of allergy and asthma is almost 20%–30% lower in patients with AD onset after 8 years of age compared to patients with AD onset before age 2 years.^14^ The median age of onset was 5 years in Cluster 1, which had 35% lower prevalence of asthma and allergy compared with Cluster 2 with a mean age of onset of 2 years. This could indicate that age of AD onset may have an impact on the risk of asthma and allergy and be useful for predicting these diseases to improve treatment and prevention targeting clinically meaningful subgroups of AD. Compared with other studies^14^, ^15^, ^16^ reporting the association between age of AD onset and the risk of asthma/allergy we have also included clinical features of AD. Cluster 1, with higher age of onset, mainly had involvement of shins, knees, and genitals. In adults, the AD lesions are frequently localized to the face and neck,^5^ as seen in Cluster 2, which also had the lowest age of onset and a high prevalence of asthma/allergy. Cluster 2 can be defined as a group of typical adults with AD. A considerable portion of patients with AD, around 30%, develops atopic hand eczema.^5^ Cluster 3 had AD lesions predominantly on their hands and feet. This cluster furthermore had the highest prevalence of asthma/allergy (90%) and an early age of onset supporting that the hand eczema in this cluster was atopic. Cluster 1 differs from the typical AD patient group with persistent AD in adulthood, as the age of onset is higher, the prevalence of other atopic diseases is lower, and AD lesions are not occurring on the typical body parts (face, neck, hands, and feet). The recognition of the less common AD subgroups is essential for proper patient management. Even though Cluster 1 has a mild disease severity it can still have a high impact on quality of life as it is localized around genitals.^17^

Our study has some limitations. First, there is an overrepresentation of women, which means that we cannot generalize results. This gender bias may also have a greater impact on results regarding genitals area. Further, the age of the included patients was around 30 years, thus not representing the older population. The entire severity spectrum of AD was also not represented based on POEM, iSCORAD and treatment, as only few from the severe category participated. Lastly, the data on other atopic diseases, age of onset, and treatment was self‐reported and could potentially be affected by recall bias. Moreover, there may also be bias associated with self‐reported data.

In conclusion, three clusters of patients with AD based on affected body areas were identified. One cluster with AD on legs and genitals, one with involvement of the upper body, and one with AD on the hands and feet. The cluster with involvement of legs and genitals was characterized by highest age of AD onset and the lowest prevalence of asthma and allergy compared to other clusters. Further, the cluster experienced subjective improvement based on POEM but was not reflected in objective measures. Understanding characteristics and longitudinal change in AD‐specific patient clusters is important to provide the best counseling and treatment.

## AUTHOR CONTRIBUTIONS

**Zarqa Ali**: Conceptualization, methodology, writing – original draft. **Kathryn Anderson**: Conceptualization, data curation, investigation, project administration, writing – review & editing. **Anders D. Andersen**: Conceptualization, project administration, supervision, writing – review & editing. **Priyanka Dahiya**: Data curation, formal analysis, software, visualization, writing – review & editing. **John R. Zibert**: Conceptualization, resource, supervision, writing – review & editing. **Simon F. Thomsen**: Conceptualization, methodology, supervision, writing – review & editing.

## CONFLICT OF INTEREST STATEMENT

Zarqa Ali and Simon F. Thomsen have no conflict of interest with regard to this paper. Anders D. Andersen, Kathryn Anderson, Priyanka Dahiya, and John R. Zibert are employed by Studies&Me.

## TRANSPARENCY STATEMENT

The lead author Zarqa Ali affirms that this manuscript is an honest, accurate, and transparent account of the study being reported; that no important aspects of the study have been omitted; and that any discrepancies from the study as planned (and, if relevant, registered) have been explained.

## Data Availability

The data that support the findings of this study are available from the corresponding author upon reasonable request.
